# Identification of nuclear export signal in KLLN suggests potential role in proteasomal degradation in cancer cells

**DOI:** 10.18632/oncotarget.27833

**Published:** 2020-12-15

**Authors:** Madhav Sankunny, Charis Eng

**Affiliations:** ^1^Genomic Medicine Institute, Lerner Research Institute, Cleveland Clinic, Cleveland, Ohio 44195, USA; ^2^Center for Personalized Genetic Healthcare, Cleveland Clinic Community Care and Population Health, Cleveland, Ohio 44195, USA; ^3^Taussig Cancer Institute, Cleveland Clinic, Cleveland, Ohio 44195, USA; ^4^Department of Genetics and Genome Sciences, Case Western Reserve University, Cleveland, Ohio 44106, USA; ^5^Germline High Risk Focus Group, CASE Comprehensive Cancer Center, Case Western Reserve University, Cleveland, Ohio 44106, USA

**Keywords:** KLLN, nuclear export, NES, proteasomal degradation

## Abstract

Germline and somatic promoter hypermethylation of *KLLN* has been found in diverse heritable and sporadic cancers, respectively. KLLN has many identified tumor suppressor functions, and when first reported, was thought to be exclusively nuclear. Here, we report on KLLN localization in both the nucleus and cytoplasm and the identification of a putative nuclear export signal (NES) sequence. KLLN overexpression in colon and breast cancer cells showed both nuclear and cytoplasmic presence. Inhibition of the CRM1 export pathway increased nuclear sequestration of KLLN, confirming the prediction of an NES sequence. Point mutations introduced in the predicted NES sequence decreased the strength of the NES and increased the nuclear sequestration of KLLN. Contrary to expectations, the transcription regulation and cellular proliferation functions of KLLN were unaffected by increased KLLN nuclear sequestration. Instead, increased nuclear KLLN correlated with increased nuclear sequestration of TRIM25 and decreased inhibitory phosphorylation of MDM2. Computational analysis of The Cancer Genome Atlas (TCGA) dataset showed positive correlation among *KLLN*, *TRIM25* and *MDM2* expression; pathway analysis of the common genes downstream of these three genes revealed protein degradation as one of the top canonical pathways. Together, our observations suggest that CRM1 pathway-based nuclear export of KLLN may impact proteasomal degradation.

## INTRODUCTION

KLLN is a tumor suppressor protein discovered in 2008 when researchers were searching for potential new targets of p53 that were involved in S-phase cell cycle checkpoint regulation. This study described KLLN as both necessary and sufficient for p53-mediated apoptosis in colon cancer cell lines [1]. The *KLLN* gene localizes to 10q23 and shares a transcription start site with *PTEN*, also a tumor suppressor gene [1, 2]. Germline mutations of *PTEN* are seen in approximately 25% of Cowden syndrome (CS) and CS-like (CSL) patients. CS is an autosomal dominant cancer predisposition syndrome with increased risks for breast, thyroid, endometrial, kidney and colon carcinomas [3, 4]. In *PTEN* mutation negative CS/CSL, *KLLN* germline promoter hypermethylation is observed in up to 35% of patients and is associated with three-fold increased prevalence of breast carcinomas and two-fold increased prevalence of renal cell carcinomas [2, 5, 6]. Recent studies looking into somatic promoter hypermethylation in ductal carcinoma *in situ* of the male breast have reported the presence of *KLLN* in a cluster of genes that are frequently methylated [7]. In oral squamous cell carcinomas, somatic *KLLN* promoter methylation was found to be associated with relapse or development of metastasis in clinical follow-up [8]. Evidence supporting somatic *KLLN* deletions have also been observed in 21% of breast carcinomas in The Cancer Genome Atlas (TCGA) and there has been an established association between increased tumor grade and decreased KLLN expression in breast carcinomas versus adjacent normal tissue [6, 9]. Therefore, KLLN could broadly contribute to both cancer susceptibility and sporadic carcinogenesis.

*KLLN* is transcriptionally regulated by p53 and has known p53-binding sites on its promoter, that when masked by the promoter hypermethylation, results in decreased KLLN expression [1, 6, 9]. Conversely, KLLN is known to regulate the expression of both *TP53* and *CHK1* by directly binding to each of its promoters [1, 6, 9]. Subsequent investigation into the high-affinity DNA binding property of KLLN using an integrative genome-wide ChIP-seq and RNA-seq analysis suggested a transcriptional regulatory function for KLLN [10]. The genome-wide ChIP-seq analysis also showed considerable enrichment for KLLN DNA binding in regions of H3K9 trimethylation. Both H3K9 trimethylation and H3K9 methyltransferase activity have been shown to be positively correlated with KLLN expression, while maintenance of pericentric heterochromatin and genetic stability negatively correlated with KLLN expression [10]. KLLN expression is also negatively associated with the regulation of cell viability as well as colony formation potential and cell migration [9, 11–13]. Some of these functions are mediated by the targeting of KLLN by microRNAs including miR-224, miR-149-3p and miR-4270 [12, 13]. *KLLN* mutations or epimutations have been reported to lead to G2 checkpoint dysfunction [6]. Furthermore, KLLN expression is negatively correlated with an appropriate response to DNA damage. This was demonstrated through the association of KLLN expression with p53 phosphorylation and acetylation after doxorubicin-induced DNA damage, and thereby, the regulation of apoptosis post-damage [14]. These studies are strongly suggestive of the role of KLLN as an important tumor suppressor protein in the cell.

The 2008 KLLN discovery study suggested that KLLN is exclusively nuclear in its cellular localization [1]. These researchers drew this conclusion based on results observed after transfection of a GFP-tagged *KLLN* plasmid in one colon cancer cell line. However, unpublished data from our lab of mass spectrometry analysis performed to identify interacting partners of KLLN revealed that a vast majority of KLLN-interacting partners and related pathways were cytoplasmic (see Results). These observations, taken together with the various putative and established functions of KLLN, suggest that KLLN should have both nuclear and cytoplasmic presence. However, since KLLN is a relatively small protein (20 kDa), it likely diffuses between the different intracellular compartments. In this study, we show that KLLN possesses a nuclear export signal (NES) that helps it exit the nucleus and that the likely role KLLN plays in trafficking proteins between the nuclear and cytoplasmic compartments may relate to proteasomal degradation.

## RESULTS

### KLLN localizes to both the nucleus and cytoplasm

**Figure 1:**
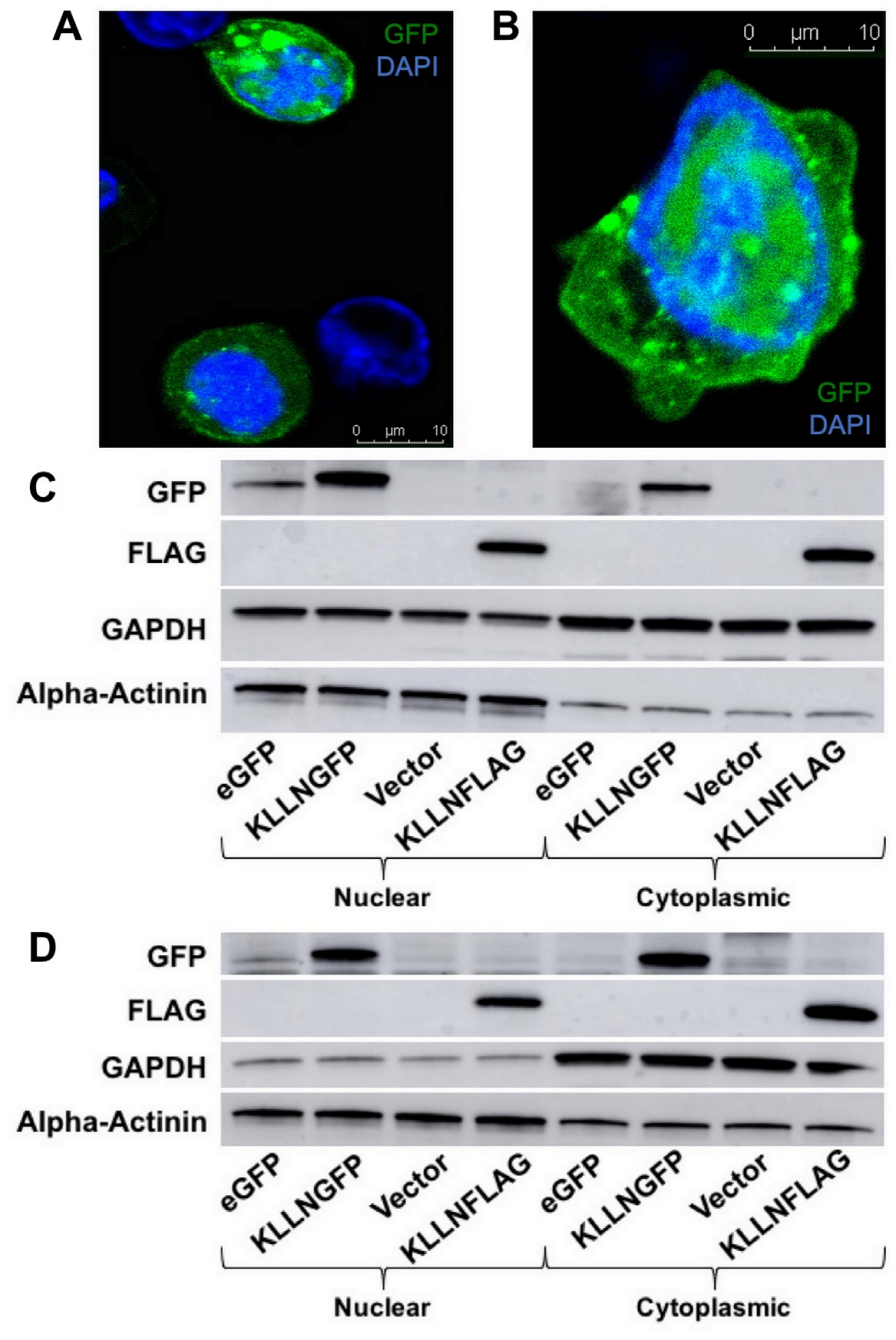
Subcellular localization of KLLN in nucleus and cytoplasm. Immunofluorescence (48 h post-transfection) in KLLNGFP overexpressing cells showed presence of KLLN in both the nucleus and cytoplasm in (**A**) HCT116 and (**B**) MCF7 cells. Immunoblotting for FLAG-tagged and GFP-tagged KLLN using FLAG and GFP antibodies respectively, in (**C**) HCT116 and (**D**) MCF7 cells confirmed the observation of KLLN presence in both nucleus and cytoplasm.

The 2008 KLLN discovery study suggested that KLLN was exclusively localized in the nucleus based on plasmid-based transfection and immunofluorescence in one colon cancer cell line [1]. Here, we show that KLLN localizes both to the nucleus and cytoplasm. The initial clues to KLLN localization to the cytoplasm were obtained from Ingenuity pathway analysis (IPA) of the top interactors of KLLN as identified by mass spectrometry. The majority of proteins that interacted with KLLN localized to cytoplasm and the top canonical pathways associated with KLLN function were also predominantly cytoplasmic (Supplementary Figure 1A and 1B). To empirically confirm the computational data, we used plasmid-based transfection of GFP and FLAG-tagged *KLLN* plasmids followed by immunoblotting to show that KLLN localizes to both the nucleus and cytoplasm in breast and colon cancer cell lines (Figure 1A and 1B). Immunofluorescence after GFP-tagged KLLN transfection confirmed that KLLN does localize to both the nucleus and cytoplasm in breast and colon cancer cell lines (Figure 1C and 1D). We were also able to validate these results in cell lines from prostate and thyroid cancers using immunoblotting and immunofluorescence (data not shown). Together, both computational and empirical data suggest that KLLN localizes to both the nucleus and cytoplasm.

### KLLN traffics between nucleus and cytoplasm regulated by the CRM-1 export pathway

**Figure 2:**
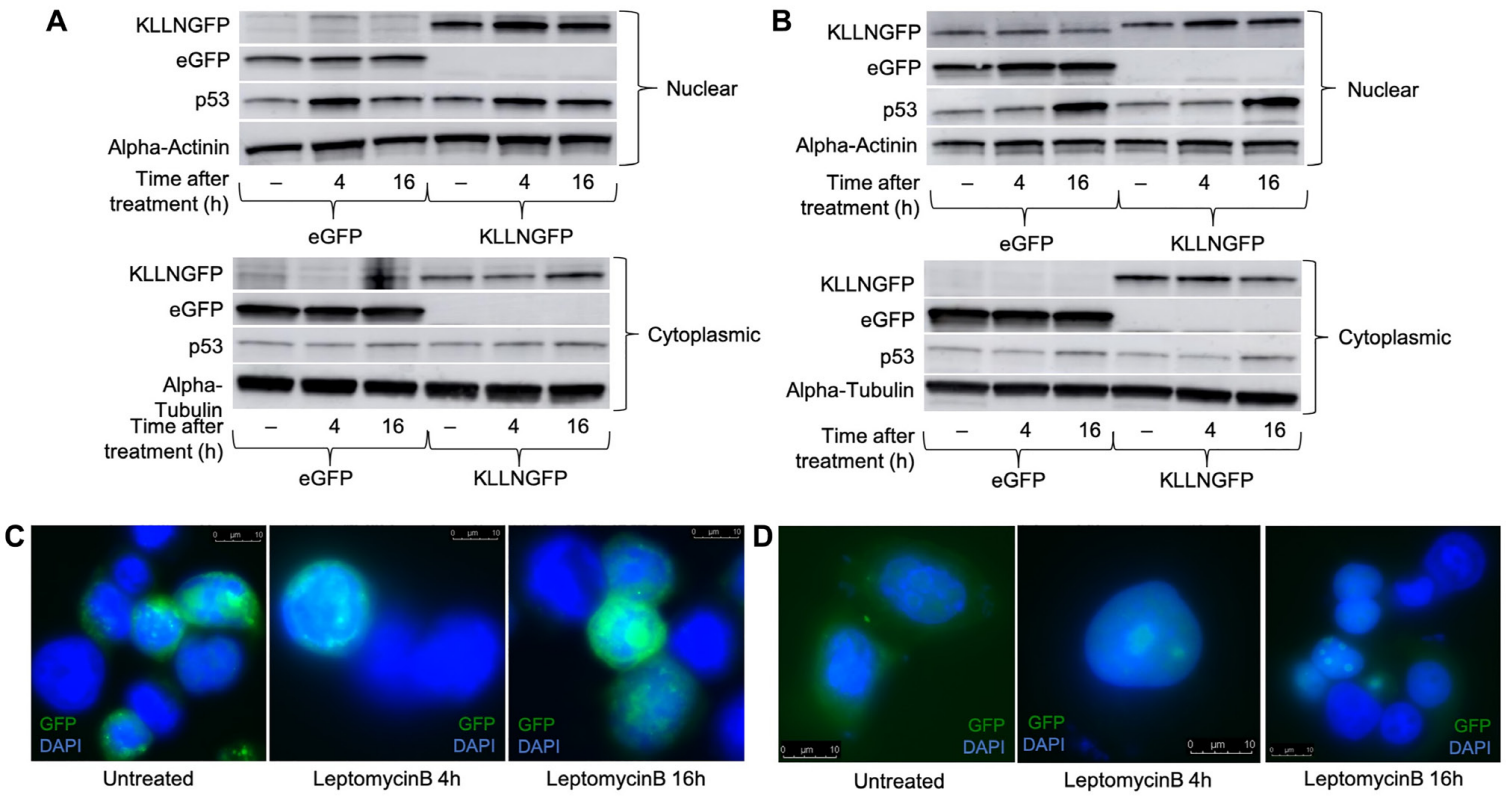
Treatment with the nuclear export blocker, leptomycin B increased the nuclear sequestration of KLLN suggesting presence of NES sequence. HCT116 and MCF7 cells transfected with either eGFP or KLLNGFP plasmids were treated with 10 nM of leptomycin B 48 h post-transfection for either 4 h or 16 h. Immunoblotting for GFP-tagged KLLN (using antibody against GFP) showed increased nuclear expression of KLLN after treatment with leptomycin B at both 4 h and 16 h time points in (**A**) HCT116, and (**B**) MCF7 cells. Increased p53 expression in the nucleus was used as a positive control for nuclear export inhibition by leptomycin B. Immunofluorescence for GFP-tagged KLLN (DAPI to stain nucleus) provided confirmation of increased KLLN sequestration following nuclear export inhibition by leptomycin B in (**C**) HCT116, and (**D**) MCF7 cells.

Since *KLLN* encodes a protein that is 20KDa, which is relatively small, it is possible that KLLN could diffuse between the two subcellular compartments. Our mass spectrometry results had suggested that some proteins that bound to KLLN were known to shuttle between the nucleus and cytoplasm, such as ribosomal protein S3 (RPS3). To investigate whether KLLN movement from the nucleus to the cytoplasm was regulated through an export pathway, we treated KLLN overexpressing cells with a known nuclear export inhibitor, Leptomycin B. Leptomycin B is a highly specific and potent inhibitor of the CRM-1 nuclear export pathway [15, 16]. Most proteins that are transported by the CRM-1 pathway that have an NES are identified by cellular mislocalization experiments done after leptomycin B treatment. Immunoblotting and immunofluorescence showed that the addition of leptomycin B increased the nuclear fraction of KLLN at both 4 h and 16 h post-treatment in colon and breast cancer cell lines (Figure 2A and 2D, Supplementary Figure 2A–2D). In HCT116 colon cells, at 16 h post-leptomycin treatment, we observed a decreased sequestration of KLLN in the nucleus by Western blotting that was confirmed by immunofluorescence (Figure 2B and 2D). Our observations suggest that KLLN movement from nucleus to cytoplasm is mediated in part by CRM-1 export pathway.

### Mutation of the putative *KLLN NES* sequesters KLLN in the nucleus

**Figure 3:**
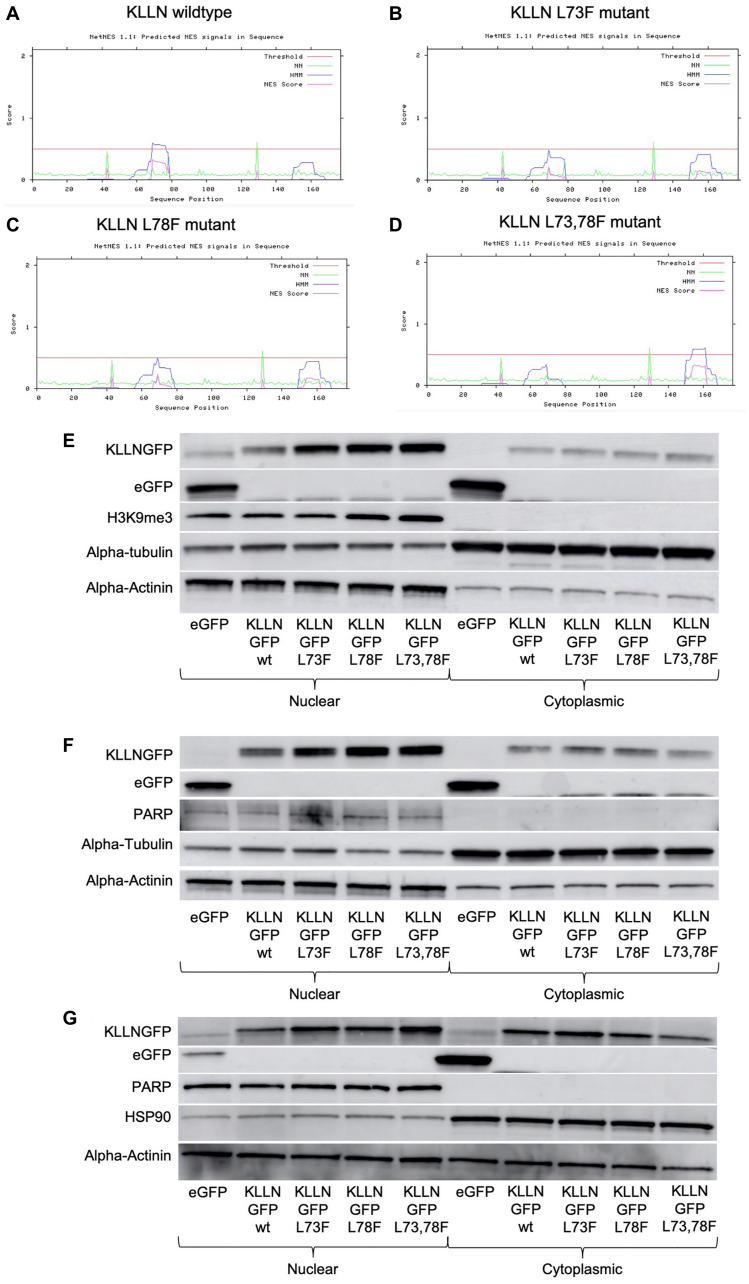
Identification of putative NES sequence on KLLN. (**A**) NetNES 1.1 prediction software identified a weak NES (sequence - LVGELSKFPL) in the KLLN protein sequence between amino acid residues 69–76. The NES on KLLN is considered weak since the predicted NES score is not greater than set threshold. (**B**–**D**) NetNES 1.1 prediction software was used to model mutations of the leucine residues at position 73 and 78 to phenylalanine residues. These mutations reduced the strength of the NES sequence on KLLN. Immunoblotting for GFP-tagged KLLN (wildtype or mutants) using antibody against GFP showed increased sequestration of the KLLN mutants in the nucleus as compared to the wildtype KLLN in (**E**) HCT116, (**F**) MCF7 and (**G**) MDA-MB-453 cells. The quantitated ratio of nuclear to cytoplasmic expression of overexpressed KLLN in each cell line was as follows: HCT116 – wt (1.0), L73F (1.8), L78F (1.9), L73_78F (1.9); MCF7 – wt (1.2), L73F (2.3), L78F (2.6), L73_78F (3.7); MDA-MB-453 – wt (1.5), L73F (2.1), L78F (2.5), L73_78F (6.0). The L73_78F mutant showed the greatest sequestration within the nucleus. PARP or H3K9me3 bands were used as controls to show efficient nuclear and cytoplasmic fractionation. Alpha actinin was used as a nuclear control for normalization and alpha-tubulin/HSP90 for cytoplasmic control.

Although proteins of small size are known to diffuse between the nucleus and cytoplasm, our observation that KLLN subcellular movement may in part be controlled by the CRM-1 export pathway led us to investigate the presence of a NES in KLLN. Using open-source prediction tools, Net NES1.1 and NUCPred, we were able to show the presence of a weak leucine-rich NES within the KLLN sequence (Figure 3A). The NES was considered weak since the predicted NES score is below the set threshold to be considered a strong NES. The predicted sequence of KLLN NES is 69’LVGELSKFPL78’. Using the prediction software, we simulated mutations of leucine 73 and leucine 78 to phenylalanines which showed decreased strength of the NES (Figure 3B–3D). In contrast, mutation of leucine 69 did not show a significant difference in NES strength. Using site-directed mutagenesis, *KLLN* plasmids were created with the L73F, L78F and L73_78F mutations. When these *KLLN* plasmids were used to overexpress mutant forms of KLLN in the colon and breast cell lines, we were able to show, using immunoblotting, that mutation of the NES resulted in increased sequestration of KLLN to the nucleus (Figure 3E–3G). We did observe a general increased KLLN expression with the mutant plasmids but the L73_78F mutant showed consistently increased nuclear sequestration in all the cell lines tested. Using ImageJ densitometric analysis, we showed that in colon cancer cell line HCT116, there was a two-fold increase in nuclear retention of the KLLN L73_78F mutant protein (Figure 3C). Similarly, in MCF7 and MDA-MB-453 cells, we observed a three-fold and four-fold increase in nuclear retention of KLLN L73_78F mutant protein (Figure 3D and 3E). PARP or H3K9me3 bands were used to confirm effective nuclear cytoplasmic fractionation indicative of the validity of the difference in sequestration of KLLN. Therefore, our results suggest that the sequence predicted as an NES is capable of mediating KLLN exit from the nucleus, to the cytoplasm.

### KLLN sequestration in the nucleus does not affect the role of KLLN in transcription regulation, or cell viability and proliferation

Previous data from our lab using direct and omics-based approaches demonstrated that KLLN plays a significant role as a transcription factor because of its DNA-binding ability [6, 9, 11]. We and others have also shown previously that KLLN regulates cellular viability and cell proliferation, thereby affirming its role as a tumor suppressor protein [9, 11–13]. Since mutations of the KLLN-NES resulted in increased sequestration of KLLN in the nucleus (above), we assessed the effects of this sequestration on established KLLN function. Quantification of gene expression of *TP53* and *CHEK1* after overexpression of the KLLN wildtype and KLLN-NES mutant proteins did not show any significant differences in the colon and breast cell lines tested (Supplementary Figure 3A–3C). We also tested expression of other genes that are known to be KLLN targets based on our previous RNA-seq and ChIP-seq data and found no significant differences in gene expression between cells with wildtype KLLN and those with NES mutant protein overexpression.

To assess if KLLN nuclear sequestration affected cell viability or cell proliferation, we performed live cell counts and MTT assays, respectively, in all our colon and breast cancer cells. We found that KLLN nuclear sequestration did not alter cell viability or cellular proliferation in any of the cell lines tested (Supplementary Figure 4A and 4B). There was no observable pattern in the minimal changes that we observed between KLLN wildtype and KLLN-NES mutant protein-expressing cells. These observations suggest that KLLN nuclear sequestration does not affect KLLN’s role in transcriptional regulation or cell viability but may play non-canonical roles.

### KLLN sequestration in the nucleus correlates with localization and activation changes of E3 ubiquitin ligases, TRIM25 and MDM2

**Figure 4:**
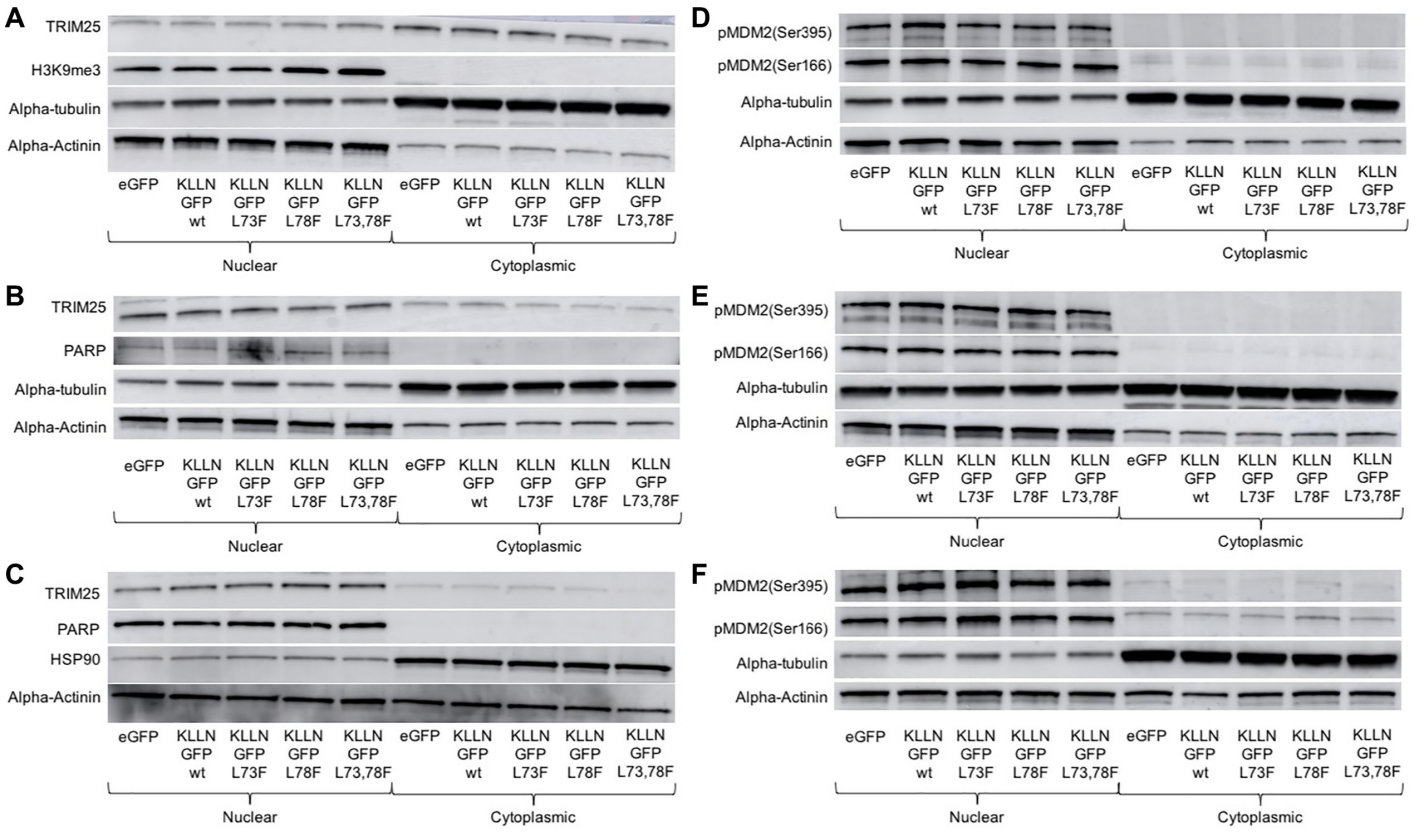
Consequences of KLLN nuclear sequestration. In (**A**) HCT116, (**B**) MCF7 and (**C**) MDA-MB-453 cells, immunoblotting for TRIM25 showed increased nuclear sequestration of TRIM25 after overexpression of mutant KLLN as compared to wildtype KLLN. In (**D**) HCT116, (**E**) MCF7 and (**F**) MDA-MB-453 cells, immunoblotting for Ser166 and Ser395 phosphorylation of MDM2 showed that the activating phosphorylation (Ser166) of MDM2 was unaffected but the inhibitory phosphorylation (Ser395) of MDM2 was decreased in cells expressing the mutant KLLN as opposed to the wildtype KLLN. PARP or H3K9me3 bands were used as controls to show efficient nuclear and cytoplasmic fractionation. Alpha actinin was used as a nuclear control for normalization and alpha-tubulin/HSP90 for cytoplasmic control.

A mass spectrometry analysis done on immunoprecipated samples after KLLN overexpression identified TRIM25, an ubiquitin E3 ligase, as one of 767 unique potential interacting partners of KLLN. TRIM25 is known to regulate p53 abundance and transcriptional activity as well as MDM2 abundance [17]. Since *TP53* gene expression is directly regulated by KLLN [9, 11], and we have also observed p53 expression changes in response to DNA damage in the absence of KLLN expression [14], we explored the consequences of KLLN nuclear retention on the subcellular localization of TRIM25. In response to increased nuclear retention of KLLN, there occurred a corresponding increase in nuclear retention of TRIM25 in colon and breast cell lines (Figure 4A–4C). Since MDM2 is also involved in the regulation of p53 levels within the cell, we also assessed the differences in phosphorylation of MDM2 at Ser166, which is an activating phosphorylation that results in oligomerization of MDM2 and ubiquitination of target proteins and at Ser395, which is an inhibitory phosphorylation. We observed that there was no effect of the increased KLLN sequestration in the nucleus on the Ser166 activating phosphorylation of MDM2, whereas the Ser395 inhibitory phosphorylation was decreased in the NES mutant (Figure 4D–4F). In all three cell lines tested, it was consistently observed that in comparison to wildtype KLLN, the NES L73,78F mutant KLLN expression resulted in the most dramatic decrease in Ser395 phosphorylation of MDM2. The expression of the other two mutant KLLN proteins showed some inconsistency in the effect on Ser395 phosphorylation of MDM2. Taken together, these results suggest that KLLN could potentially play a role in proteasomal degradation of proteins and that the nuclear export of KLLN is likely an important step in this function.

### Presence of NES on KLLN suggests role in trafficking proteins into the cytoplasm for degradation

**Figure 5:**
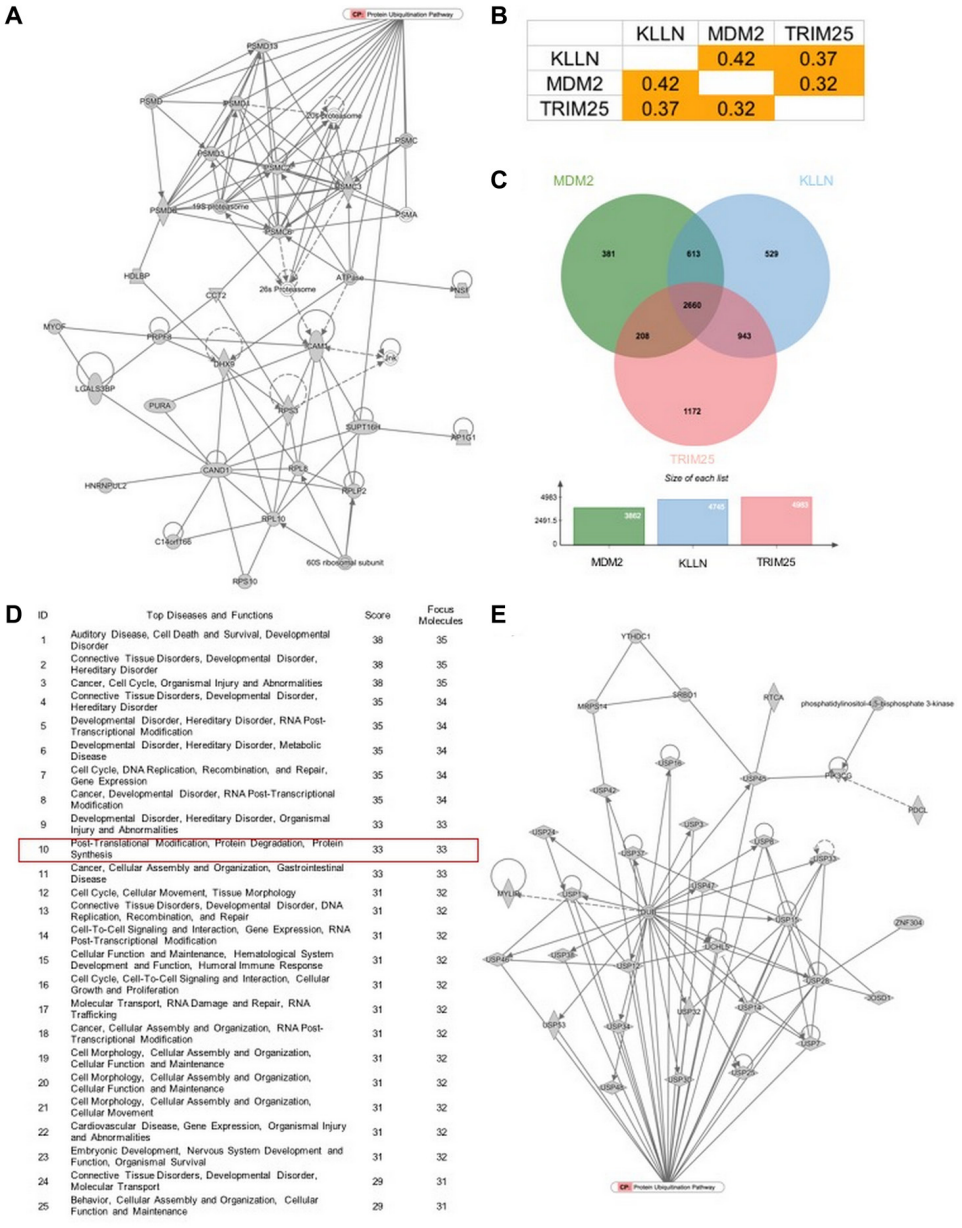
KLLN function correlates with proteasomal degradation. (**A**) IPA-based pathway analysis image of direct interactors of KLLN identified by mass spectrometry involved in protein degradation pathway. (**B**) Positive correlation of gene expression of KLLN, TRIM25 and MDM2 in renal cell carcinoma data collected from TCGA. (**C**) A Venn diagram of positively correlated genes associated with KLLN, TRIM25 and MDM2 show more than half of positively-correlated genes were common to KLLN, TRIM25 and MDM2. (**D**) IPA-based pathway analysis showing protein degradation as a top 10 pathway associated with the common genes positively correlated with KLLN, TRIM25 and MDM2. (**E**) Graphical representation of the contribution of the common positively correlated genes in protein degradation/proteasomal degradation.

Mass spectrometry-based proteome analysis with IPA revealed KLLN-interacting proteins predicted to play roles in protein degradation pathways (Figure 5A). Our data confirm the relationship of KLLN with common E3 ubiquitin ligases. To gather more evidence towards KLLN involvement in protein degradation, we used computational analysis of publicly available data from TCGA using the UALCAN web resource [18] and observed that in renal cell carcinomas, KLLN, TRIM25 and MDM2 gene expression are positively correlated with each other (Figure 5B). We also used the program ‘jvenn’ [19] to generate Venn diagrams of common genes positively or negatively correlated with KLLN, TRIM25 and MDM2 and showed that there was considerable overlap among the genes that were correlated with each of our three input genes (Figure 5C). We then analyzed the common list of positively correlated genes using the IPA tool to identify connected pathways and revealed that the most relevant pathways were all involved in cellular activities related to organismal development and injury, with one of the top pathways being post-translational modification and protein degradation (Figure 5D and 5E). Taken together, all of our observations suggest that KLLN may play a sentinel role in the trafficking of proteins to the cytoplasm for proteasomal degradation.

## DISCUSSION

The subcellular localization of KLLN as a nuclear protein that tends to concentrate in the nucleolar region was established in the discovery study observing overexpressing GFP-tagged KLLN in the colon cancer cell line HCT116. This was considered conventional wisdom given that subsequent studies revealed KLLN’s role in transcription and genome stability [6, 9–11]. However, our empiric analysis of the proteins that were identified as interacting partners of KLLN by mass spectrometry suggested that a majority of these proteins were present in the cytoplasm and involved pathways related to EIF2 signaling, protein ubiquitination, and caveolar-mediated endocytosis. Our current study establishes the presence of KLLN in the nucleus and cytoplasm. Based on the prediction of a potential NES sequence in KLLN, we explored the exportin/importin-regulated movement of KLLN between the sub-cellular compartments and were able to demonstrate that KLLN indeed possessed a NES sequence that regulated the export of the protein through the CRM-1 pathway. Even though mutations in the putative NES sequence increased the nuclear sequestration of KLLN, it did not affect KLLN’s regulation of transcription or cellular proliferation. Instead, we identified a potential role for KLLN in proteasomal degradation where the nuclear export of KLLN may be an essential regulatory step.

Since KLLN interacts with proteins that were known to be involved in active trafficking between the cytoplasm and the nucleus, we hypothesized that even though KLLN is a small protein, it may have a nuclear export signal (NES) or nuclear localization signal (NLS). Conventional wisdom suggests proteins of small size diffuse between the different subcellular compartments but there are examples of proteins of small size with either a NES or NLS sequence that use the export or import complexes to move between the various subcellular compartments such as RPS3. Standard experimental evidence for CRM1-mediated nuclear export of a protein includes sensitivity of the cargo protein to nuclear export inhibition mediated by leptomycin B [20]. Here, we show that exposure to leptomycin B led to increased sequestration of KLLN in the nucleus at both early and late timepoints, suggesting that the nuclear export of KLLN is through the CRM1 export pathway. There have been suggestions in the past that consensus-based prediction does not pick up all experimentally identified NESs and alternatively identifies false positive sequences [20–24]. To identify the putative NES sequence on KLLN, we initially used a consensus sequence prediction software and identified a weak sequence. The NES sequence identified in the KLLN protein is between residues 69 and 78 and reads LVGELSKFPL. This sequence fits one of the commonly accepted NES consensus sequences Φ1-X_3_– Φ2-X_2_– Φ3-X– Φ4. This consensus sequence has a predominance of leucine residues in the hydrophobic regions marked by Φ as does the identified sequence on KLLN suggesting that the NES identified might be a true sequence.

It is accepted that the computational identification of a NES sequence has to be experimentally validated either through cellular mislocalization after leptomycin B treatment or nuclear export assays in cells. So, based on our results showing KLLN nuclear export was sensitive to leptomycin B and the predicted NES conforming to a general consensus sequence, we believe that the sequence identified in KLLN is a strong candidate for NES. Another interesting observation is that the identified NES sequence falls within one of two phylogenetically highly conserved regions. Based on mutations observed in patients, we mutated residues 73 and 78 that are also part of our NES consensus sequence. The prediction software confirmed that these mutations did reduce the strength of the NES. Experimental evidence also confirmed the reduced strength of the NES based on the observation that the overexpressed mutant KLLN protein sequestered in the nucleus more as compared to the wildtype KLLN. Taken together, these findings and observations gave us confidence that even though the predicted strength of the NES in KLLN was weak, this was a true NES sequence.

Given our confidence in KLLN’s NES, we wished to explore its role. The logical path of reasoning would be affecting KLLN’s canonical function in regulation of transcription and cellular proliferation. Surprisingly, the increased nuclear sequestration of KLLN did not affect either of these functions. One speculation of this paradox would suggest that KLLN’s canonical nuclear function of transcription with subsequent cellular proliferation is based on threshold, and not on increasing gradients. By turning to our previous mass spectrometry data, we were able to identify TRIM25 as a direct interactor of KLLN. TRIM25 is a known regulator of p53 and MDM2 abundance and inhibits the transcriptional activity of p53 in response to DNA damage [17]. Overexpression of TRIM25 is also known to promote cell proliferation and survival in prostate cancer [25]. Previous results that suggest the presence of a regulatory loop between p53 and KLLN motivated us to explore the role of a KLLN-TRIM25 interaction in connection with p53 and MDM2 regulation. The increased sequestration of mutant KLLN in the nucleus was also associated with an increased nuclear sequestration of TRIM25 and decreased inhibitory phosphorylation of MDM2 (Ser395). The regulation of TRIM25 and MDM2 by KLLN could also explain the observations from our previous studies looking into the p53-KLLN regulatory axis. In this specified study, we had observed that knocking down KLLN reduced the response of p53 to DNA damage induced by doxorubicin, specifically the activation and expression of p53 [14]. A lack of KLLN expression in the cell would therefore give free rein to TRIM25 and MDM2 in their inhibitory effects on p53 expression and activation resulting in the dysregulation of the DNA damage response pathway as observed.

*In silico* analysis of the TCGA dataset using the UALCAN web resource identified a KLLN-TRIM25-MDM2 regulatory axis in renal cell carcinoma. There was an overabundance in overlap of genes whose expression was positively correlated with the expression of each of the three genes, *KLLN*, *TRIM25* and *MDM2*. Pathway analysis using IPA revealed that one of the top canonical pathways was protein degradation, as expected. Our previous mass spectrometry results for direct interactors of KLLN had also revealed an overabundance of proteins involved in proteasomal degradation. Taken together, the *in vitro* and *in silico* data suggest that the nuclear export of KLLN has a potential role in protein degradation. Unfortunately, similar analysis performed on breast and colon adenocarcinoma TCGA datasets did not show comparable correlation results. This was attributable to the incomplete or short list of *MDM2* correlated genes in both breast and colon cancer. Therefore, the number of representative genes in the shared gene lists was not sufficient to do a pathway analysis. However, for the same two cancers, when a pathway analysis was run on the shared list of genes whose expression correlates positively with *KLLN* and *TRIM25*, the protein ubiquitination pathway was observed to be the top canonical pathway. Since renal cell carcinoma is a component cancer of Cowden syndrome and KLLN is a known cancer predisposition marker for CS/CSL patients, we used the renal cell carcinoma data as a surrogate to identify the KLLN-TRIM25-MDM2 axis in the regulation of protein degradation.

A technical limitation of our study is the inability to detect native KLLN protein due to the lack of reliable KLLN antibodies [14, 26]. None of the commercially available KLLN antibodies have passed our intense quality control protocol. Most of these antibodies are unable to detect FLAG or GFP-tagged KLLN protein and in some cases, the antibodies showed reactivity to mouse or rat samples, even though it is well established that KLLN has no rat or mouse homolog. The lack of a high specificity KLLN antibody is due to the difficulty of purifying the KLLN protein. This difficulty is due to the conformation adopted by KLLN expressed in bacteria that is known to have very low affinity for purification columns and therefore, makes the purification of the native protein close to impossible [1, 27]. Another factor that makes the purification of KLLN challenging is that the protein has a large number of predicted trypsin cleavage sites and therefore, is easily fragmented during purification. To circumvent this technical limitation, in our study we tagged KLLN with either a GFP tag or a FLAG tag and used antibodies against the tags to detect the protein expression and localization of KLLN.

In conclusion, our results identifying the putative NES sequence of KLLN establishes KLLN as a cargo protein for the CRM1 export pathway complex. Further, the association between KLLN localization and proteasomal degradation establishes a potential new function for KLLN. Even though TRIM25 is a known regulator of p53 and MDM2 expression and activity, our study establishes a correlative role for KLLN in this pathway. In our previous study, we had suggested the closing of a regulatory loop between p53 and KLLN in response to DNA damage and this study has contributed the missing pieces to that regulatory loop.

## MATERIALS AND METHODS

### Cell culture

Cell culture was done according to the protocol standardized by our lab [10]. HCT116 colon cancer cells, and MCF7 and MDA-MB-453 breast cancer cells were cultured in DMEM media supplemented with 10% FBS (Life Technologies, Carlsbad, CA, USA). Cell lines were cultured at 37°C and 5% CO_2_ and passaged using Trypsin-EDTA. All cell lines were purchased from ATCC (Manassas, VA, USA) after 2010 and authenticity was documented by standard STRS analysis per ATCC routine. All cell lines were used during passage 3 to 20 and routinely tested for mycoplasma.

### Overexpression of KLLN by plasmid transfection

The eGFP plasmid backbone was sourced from Clontech (Mountain View, CA, USA). The wildtype KLLN plasmids were created in house using the eGFP backbone with *KLLN* sequence cloned in either the C-terminal or the N-terminal side of eGFP sequence. Plasmids with predicted *KLLN*-NES mutations were created using the QuikChange II kit (Agilent Technologies, Inc., Santa Clara, CA, USA). For transfection of the different plasmids, cells were seeded at 60–70% confluence in appropriate dishes and allowed to attach overnight. For overexpression of KLLN, cells were transfected with either the wildtype *KLLN* plasmid or the *KLLN*-NES mutant plasmids using Lipofectamine LTX (Life Technologies) or Lipofectamine 3000 (Life Technologies) according to the manufacturers protocol. Empty eGFP vector transfected cells were used as a control. Cells were collected for analysis 48–72 h after transfection. qRT-PCR and Western blotting were used to confirm transcript and protein overexpression of KLLN.

### Leptomycin B treatment

Leptomycin B (cat# L2913, Sigma-Aldrich, Burlington, MA) was used to inhibit nuclear export through the CRM1 export pathway in the breast and colon cancer cell lines. A 10 nM dose of leptomycin B was considered as the appropriate dose for our studies. Leptomycin B addition 48–72 h post-transfection with empty vector or KLLN plasmid was considered appropriate. Multiple timepoints for leptomycin B treatment was tested, and 4 and 16 h were chosen for our studies.

### RNA collection, reverse transcription, and quantitative PCR

RNA collection, reverse transcription and quantitative PCR was done according to the protocol standardized by our lab [10, 14]. Total RNA was collected using the Nucleospin RNA plus kit (Takara Bio USA, Mountain View, CA, USA) and RNA concentration quantified using a Nanodrop spectrophotometer (Thermo Fisher Scientific, Waltham, MA, USA). Reverse transcription was done using Primescript RT reagent kit (Takara Bio, USA) following the manufacturers protocol. Complementary DNA (cDNA) was quantified using SYBR Green PCR Master Mix (Life Technologies) on the 7500 Real Time PCR System (Applied Biosystems, Foster City, CA, USA) using primers specific for KLLN and GAPDH. Data was analyzed using the standard 2^-ΔΔCT^ method.

### Protein extraction and quantification

For whole cell lysate preparation, cells harvested after transfection and/or doxorubicin treatment were lysed using Mammalian Protein Extraction Reagent (Pierce, Rockford, IL, USA) supplemented with protease and phosphatase inhibitors (Sigma-Aldrich) at 4°C for 30 min - 1 h. Lysates were collected from cell debris by centrifugation at 16,000 × g for 5 mins.

For nuclear and cytoplasmic fractionation, we used a modified protocol from Gagnon et al. [28]. Cells were harvested after transfection and/or doxorubicin treatment and the cytoplasmic fraction was isolated by incubating cells in 100–200 μl (based on size of cell pellet) of HLB buffer for 10 mins at 4°C and spinning down at 800 × g for 8 min at 4°C. to the collected cytoplasmic fraction 3–6 μl of 5M sodium citrated is added, mixed well and set aside at 4°C. The nuclear pellet is washed with 500 μl of HLB buffer after which the nuclear fraction is isolated using 50–100 μl (half the volume of HLB buffer used for cytoplasmic fractionation) and sonicated at 4× strength for 5 secs each 3 times on a sonicator (Sonic Dismembrator, Model 100, Fisher Scientific, Hampton, NH, USA). After sonication, both the nuclear fraction and cytoplasmic fraction are spun down at 16,000 × g at 4°C and lysates collected and stored at –80°C.

Protein concentrations were quantified using the BCA Protein Assay (Pierce) according to manufacturer’s instructions. Concentration of the lysates were normalized to 1–2 mg/ml using 2X Laemmli dye (Sigma-Aldrich) and was heated at 100°C for 10 min and prepared for western blotting.

### Western blotting

Western blotting was done according to the protocol standardized by our lab [14]. Approximately 20–40 μg of normalized protein was run on a Criterion™ 4–15% gradient gel (Bio-Rad, Hercules, CA, USA). Fractionated protein was transferred to a nitrocellulose membrane using Trans-Blot Turbo (Bio-Rad) according to manufacturer’s instructions and membranes blocked for 30 min – 1 h in 3% BSA. Membranes were incubated overnight with primary antibody at 4°C. Blots were washed the next day using 1X TBST (3 times, 7 mins each), incubated with secondary antibody for 1 h at room temperature and washed again with 1X TBST (3 times, 7 mins each). Superscript West Pico chemiluminescent substrate (Thermo Fisher Scientific) or Clarity Western ECL substrate (Bio-Rad) were used for chemiluminescent detection and images were captured using Amersham Imager 600 (GE Healthcare Sciences, Marlborough, MA, USA). Densitometric analysis was done using the NIH software ImageJ.

Primary antibodies commonly used in this study were against p53 (1:1000) (cat# sc126) GFP-B2 (1:1000) (cat# sc9996) and TRIM25 (1:1000) (cat# sc166926) from Santa Cruz Biotechnology, Santa Cruz, CA, USA, and FLAG (1:1000) (Sigma-Aldrich, cat# F-1804) and H3K9me3 (1:1000) (Epigentek, Farmingdale, NY, USA). Antibodies against pMDM2 (Ser166) (1:1000) (cat# 3521), GAPDH (1:20000) (cat# 2118), PARP (1:1000) (cat# 9542), HSP90 (1:1000) (cat# 4874) were sourced from Cell Signaling, Danvers, MA, USA. Alpha-actinin (1:1000) (Cell Signaling, cat# 3134) and alpha-tubulin (1:10000) (Sigma-Aldrich, cat# T6074) were used as loading controls. Secondary antibodies used were Anti-mouse HRP and anti-rabbit HRP (1:2500) (Promega, Madison, WI, cat#s W4021, W4011).

### Immunofluorescence

Immunofluorescence was done according to the protocol standardized by our lab [14]. For immunofluorescence, cells were grown on cover slips in a 6-well plate and transfected with *KLLN* plasmids. Seventy-two hours after transfection, cells were treated with the appropriate dose of leptomycin B or left untreated. After specific intervals of times with treatment, cells on the coverslips were washed with 1X PBS and fixed with 3.7% formaldehyde for 15 minutes at room temperature. After washing with 1X PBS, cells were permeabilized in 0.3% Triton-X in 1X PBS for 5 minutes at room temperature. After washing, coverslips were mounted onto slides with DAPI-containing mounting media (Vector Laboratories, Burlingame, CA, USA). Slides were blinded and images were analyzed using upright confocal microscopy (Leica Microsystems, Buffalo Grove, IL, USA) and Leica Confocal Software for image analysis.

### Cell viability assay

To assess cell viability, cells were grown in 6-well plates, and transfected with empty vector, and wildtype *KLLN* and *KLLN*-NES mutant plasmids. Cells were harvested 48–72 h after transfection and counted on a Countess™ automated cell counter (Thermo Fisher Scientific) according to the manufacturers protocol. The number of viable cells were enumerated and plotted on a graph.

### Cell proliferation assay (MTT assay)

Cells were grown in 6-well plates, and transfections were done as described for cell viability assay. After the appropriate intervals of times with treatment, 100 μl of MTT reagent (5 mg/ml) was added to each well containing 1 ml of fresh media. After incubation at 37°C for 1 h, the media was removed and 1 ml of DMSO was added to each well. After another incubation at 37°C for 10 min, the DMSO was mixed and three 200 μl aliquots per well were transferred to a 96-well plate. Absorbance is read at 560 nm and average absorbance values were plotted on a graph.

### Statistical analysis

All experiments were performed in duplicate or triplicate. All data were represented with error bars signifying standard error of mean for population mean and standard deviation for sample mean. If relevant, data collected from these *in vitro* studies were analyzed by two-tailed Student’s *t*-tests. Significance of results was determined based on calculated *p*-values and a *p*-value less than 0.05 was considered statistically significant.

## SUPPLEMENTARY MATERIALS


