# Supplementary material for: Identification of nuclear export signal in KLLN suggests potential role in proteasomal degradation in cancer cells
Source: Oncotarget. 2020 Dec 15;11(50):4625–36. doi: 10.18632/oncotarget.27833 (PMC7747863; doi:10.18632/oncotarget.27833)
Supplement: Supplementary file 1 [file oncotarget-11-4625-s001.pdf]

# Identification of nuclear export signal in KLLN suggests potential role in proteasomal degradation in cancer cells

## SUPPLEMENTARY MATERIALS

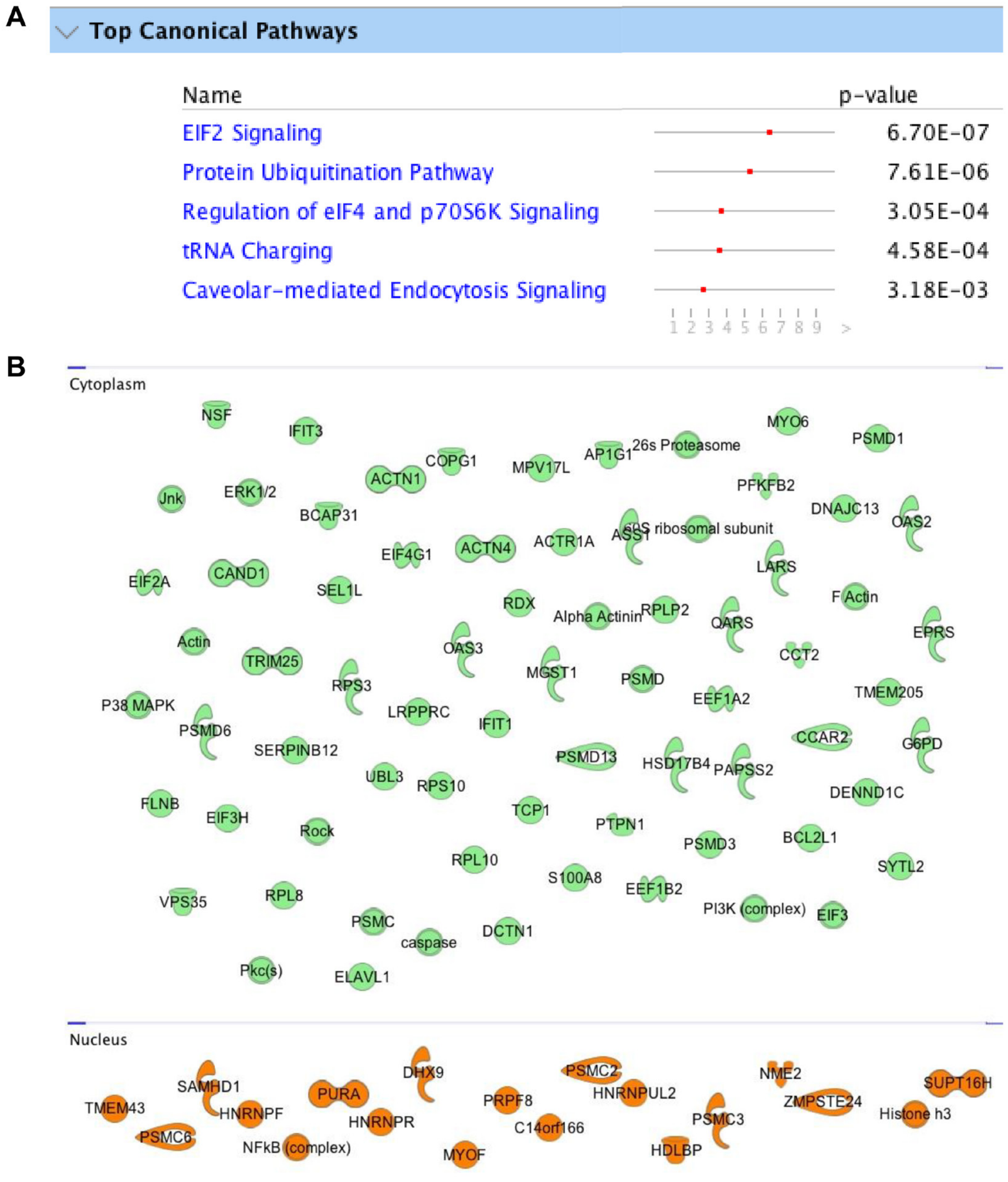


**Supplementary Figure 1:** Ingenuity pathway analysis (IPA) of top interactors of KLLN identified by mass spectrometry show (**A**) the top canonical pathways associated with interacting partners of KLLN to be predominantly cytoplasmic and (**B**) the majority of the proteins that interact with KLLN to be localized in the cytoplasm.

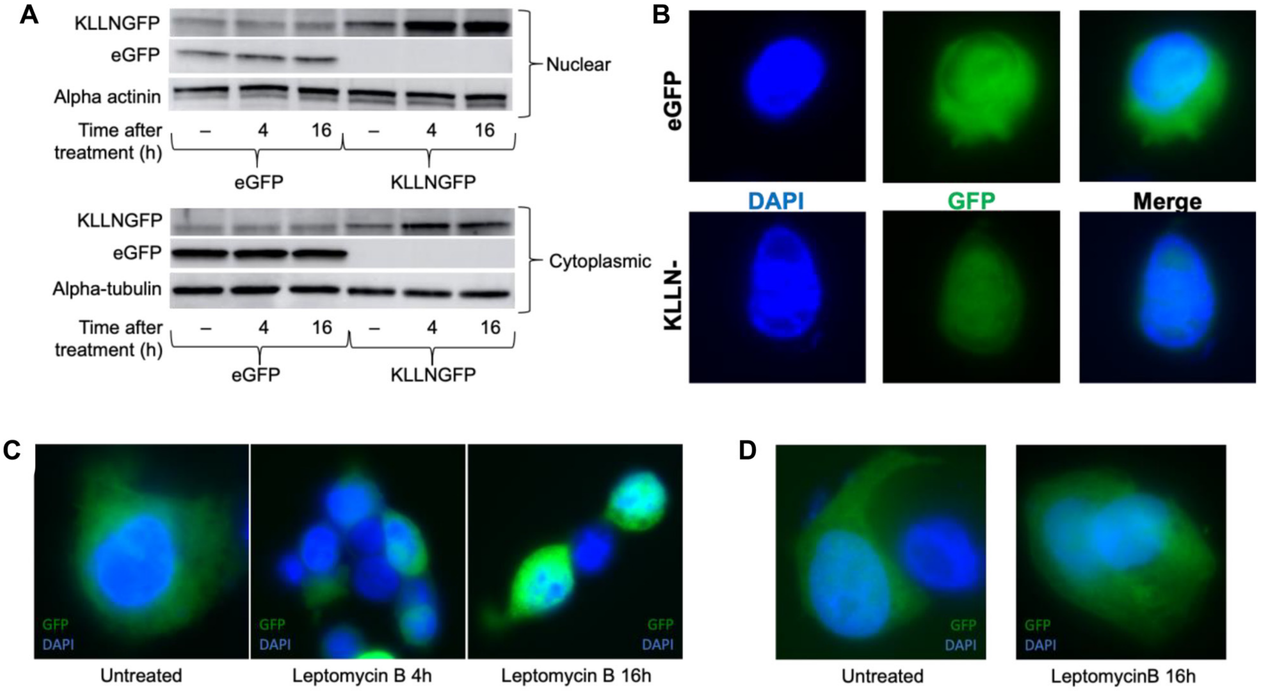


**Supplementary Figure 2: Inhibition of nuclear export using leptomycin B increased the nuclear sequestration of KLLN.** (**A**) Immunoblotting for GFP-tagged KLLN in MDA-MB-453 cells showed increased nuclear sequestration at 4 h and 16 h after addition of leptomycin B. No change was observed on eGFP localization after treatment with leptomycin B. (**B**) Immunofluorescence for GFP-tagged KLLN in MDA-MB-231 cells showed increased nuclear sequestration at 16 h after addition of leptomycin B but eGFP localization was unaffected in (B) MDA-MB-231, (**C**) HCT116 and (**D**) MCF7 cells.

**A**

| | KLLN CDS | KLLNintron | CHK1 | TP53 |
|---|---|---|---|---|
| eGFP | 1.0 | 1.0 | 1.0 | 1.0 |
| KLLNwt | 9186.5 | 1.4 | 0.8 | 1.0 |
| KLLNL73F | 11243.8 | 1.0 | 0.8 | 0.8 |
| KLLNL78F | 11889.3 | 0.8 | 1.0 | 0.8 |
| KLLNL7378F | 13871.3 | 0.8 | 1.3 | 0.3 |

**B**

| | KLLN CDS | KLLNintron | CHK1 | TP53 |
|---|---|---|---|---|
| eGFP | 1.0 | 1.0 | 1.0 | 1.0 |
| KLLNwt | 1489.9 | 1.0 | 0.9 | 1.0 |
| KLLNL73F | 1747.3 | 1.1 | 1.0 | 1.2 |
| KLLNL78F | 3045.6 | 0.9 | 0.9 | 1.0 |
| KLLNL7378F | 1317.3 | 1.0 | 0.9 | 0.9 |

**C**

| | KLLN CDS | KLLNintron | TP53 |
|---|---|---|---|
| eGFP | 1.0 | 1.0 | 1.0 |
| KLLNwt | 1207.5 | 1.0 | 1.0 |
| KLLNL73F | 923.0 | 1.2 | 1.0 |
| KLLNL78F | 1849.3 | 1.0 | 1.0 |
| KLLNL7378F | 823.6 | 0.8 | 1.1 |

**Supplementary Figure 3:** Overexpression of wildtype or NES mutant *KLLN* showed no significant difference in *TP53* or *CHK1* expression in (**A**) HCT116, (**B**) MCF7 and (**C**) MDA-MB-453 cells. Primers specific for *KLLN* coding sequence (CDS) and intron showed that overexpression due to plasmid-based transfection did not affect the genomic expression of *KLLN*.

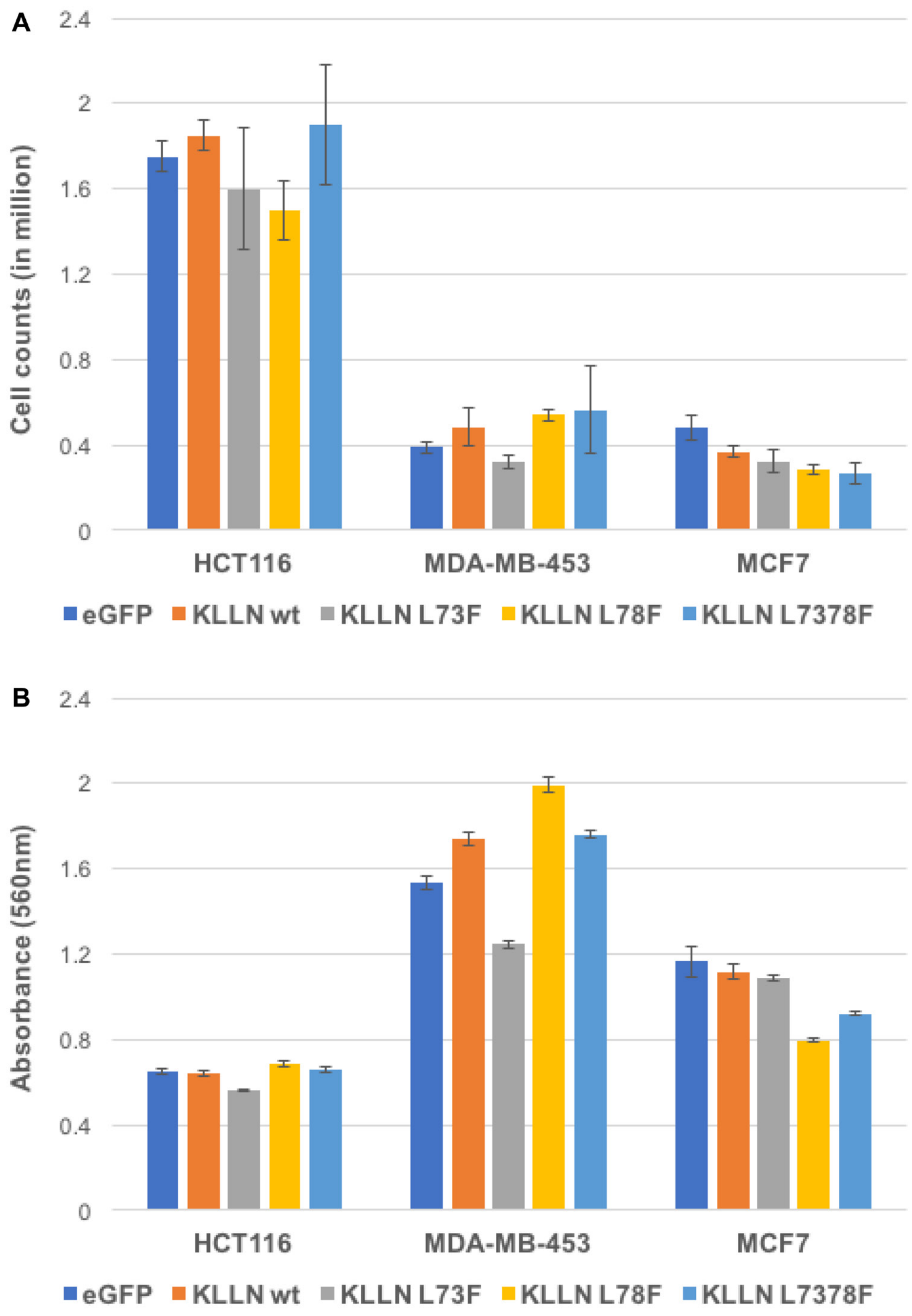


**Supplementary Figure 4:** Increased nuclear sequestration of KLLN due to mutation of the NES sequence shows no effect on (**A**) cell viability as measured by cell counts and (**B**) cell proliferation assessed by MTT assay. There was no significant difference observed between wildtype KLLN expression and NES mutant KLLN expression on cell counts or absorbance reading for MTT assay in HCT116, MDA-MB-453 and MCF7 cells.
